# Urolithin A attenuates auditory cell senescence by activating mitophagy

**DOI:** 10.1038/s41598-022-11894-2

**Published:** 2022-05-11

**Authors:** Sung Il Cho, Eu-Ri Jo, Hansoo Song

**Affiliations:** 1 Department of Otolaryngology-Head and Neck Surgery, Chosun University College of Medicine, 365 Pilmun-daero, Dong-gu, Gwangju, 61453 South Korea; 2 Department of Occupational and Environmental Medicine, Chosun University College of Medicine, Gwangju, South Korea

**Keywords:** Neuroscience, Auditory system

## Abstract

Aging of sensory organs is associated with a decline in mitochondrial function and the accumulation of dysfunctional mitochondria. Impaired mitophagy blocks the turnover of dysfunctional mitochondria and leads to their accumulation. Urolithin A (UA) induces mitophagy in various mammalian cells. This study was aimed at investigating the effect of the mitophagy activator, UA, on premature senescent auditory cells. The levels of cellular senescence-associated p53 and p21 significantly increased in H_2_O_2_-induced senescent House Ear Institute‐Organ of Corti 1 (HEI-OC1) cells and cochlear explants. However, the levels of mitophagy-related molecules significantly decreased. UA significantly decreased the expression of senescence-associated p53 and p21, and increased the expression of mitophagy-related proteins, in H_2_O_2_-induced senescent cells and cochlear explants. The percentage of β-galactosidase-stained senescent cells also reduced in H_2_O_2_-treated cells and cochlear explants upon UA pre-treatment. The formation of mitophagosomes and mitophagolysosomes was restored upon UA pre-treatment of H_2_O_2_-induced senescent cells. The knockdown of mitophagy-related genes (*Parkin* and *Bnip3*) resulted in annulment of UA-induced anti-senescent activity. UA significantly increased the ATP content, mitochondrial DNA (mtDNA) integrity, and mitochondrial membrane potential in senescent HEI-OC1 cells. These findings indicate that UA counteracted mitophagy decline and prevented premature senescence in auditory cells. Hence, UA administration might be a promising strategy for preventing mitochondrial dysfunction in patients with age-related hearing loss.

## Introduction

Aging occurs in all sensory organs, and cellular senescence plays an important role in this process^1^. Premature senescence of auditory cells can be induced by oxidative stress and can result in hearing loss^2^. Mitochondrial dysfunction is one of the hallmarks of the aging process^3^ and is associated with the development of age-related neurodegenerative diseases^4^. Aging mitochondria show morphological alterations and decreases in mitochondrial DNA (mtDNA) content, protein levels, and organelle mass^5^.

Autophagy is a homeostatic process by which damaged or dysfunctional cellular components are removed through the formation of autophagosomes, which sequester the cellular components and then fuse with lysosomes^6^. Autophagy enables cells to recover from stress, such as oxidative and endoplasmic reticulum stress, and energy depletion^7,8^. Impairing autophagy through the knockdown of autophagy-related genes induces premature senescence in human fibroblasts^9^. Mitochondrial selective autophagy, termed mitophagy, specifically degrades dysfunctional or damaged mitochondria and maintains a healthy mitochondrial population^10^. Urolithin A (UA) is a natural food metabolite that is abundant in strawberries, pomegranates, and nuts^11^. The role of UA as an activator of mitophagy in both mice and humans has been investigated^11,12^. It prevents the accumulation of damaged or dysfunctional mitochondria and has a favorable safety rating according to standardized toxicology tests^13^.

Cellular senescence induced by oxidative stress has been used as an in vitro model for aging research^14^. Improving mitochondrial function by inducing mitophagy is a promising approach to delay or halt the development of senescence^12^. We hypothesized that reactivation of mitophagy using UA might attenuate senescence-inducing stress of auditory cells. This study was aimed at investigating the effect of UA on oxidative stress-induced senescence in auditory cells.

## Results

### Oxidative stress reduces levels of mitophagy-related proteins and induces premature senescence in House Ear Institute‐Organ of Corti 1 (HEI-OC1) cells and cochlear explants

**Figure 1:**
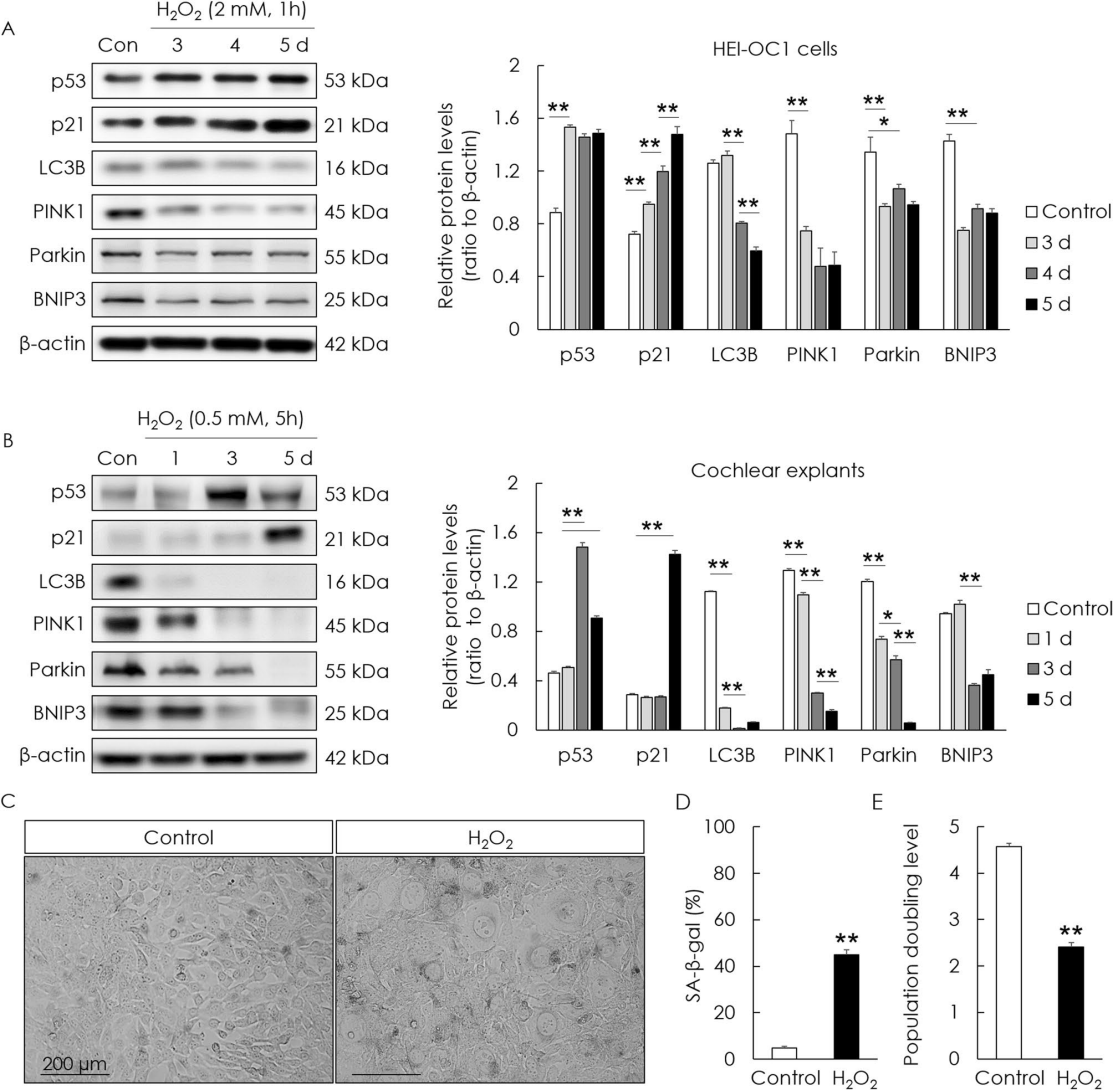
H_2_O_2_-induced senescence and changes in mitophagy-related proteins in HEI-OC1 cells and cochlear explants. HEI-OC1 cells were exposed to 2 mM H_2_O_2_ for 1 h and incubated for 3, 4, and 5 days. Whole cochleae also were exposed to 0.5 mM H_2_O_2_ for 5 h and incubated for 1, 3, and 5 days. The levels of p53 and p21 (cellular senescence markers) increased in H_2_O_2_-treated HEI-OC1 cells (**A**) and cochlear explants (**B**). The levels of mitophagy-related proteins (PINK1, Parkin, BNIP3, and LC3B) significantly decreased in H_2_O_2_-treated HEI-OC1 cells (**A**) and cochlear explants (**B**). HEI-OC1 cells were exposed to 2 mM H_2_O_2_ for 1 h and incubated for 5 days. β-galactosidase staining of senescent cells significantly increased in H_2_O_2_-treated cells (**C**, **D**). The population doubling level significantly decreased in H_2_O_2_-treated cells (**E**). Data are presented as means ± standard errors of the mean of five independent experimental results (SA-β-gal, senescence associated-beta galactosidase). *p < 0.05, **p < 0.01. The grouping of gels/blots cropped from different parts of the same gel. Full-length blots are presented in Fig. S1A, S1B.

To investigate premature cellular senescence and the changes in mitophagy-related proteins upon the application of H_2_O_2_ to HEI-OC1 cells and cochlear explants, senescence-associated p53 and p21, and mitophagy-related proteins, were examined. The levels of cellular senescence-associated p53 and p21 significantly increased in H_2_O_2_-treated cells and cochlear explants. However, the levels of mitophagy-related proteins—PINK1, Parkin, BNIP3, and LC3B—significantly decreased (Fig. 1A,B). Cellular senescence was examined using β-galactosidase activity; senescence was induced in HEI-OC1 cells 5 days after H_2_O_2_ treatment at a rate of 44.9% ± 2.1% (Fig. 1C,D). The population doubling rate indicates the speed of cell growth. The population doubling rate of H_2_O_2_-treated cells decreased (2.41 ± 0.1) compared with that of normal cells (4.57 ± 0.1; Fig. 1E). These results demonstrated that H_2_O_2_ treatment induced cellular senescence and reduced the levels of mitophagy-related proteins in auditory cells.

### UA counteracts mitophagy decline and decreases cellular senescence

**Figure 2:**
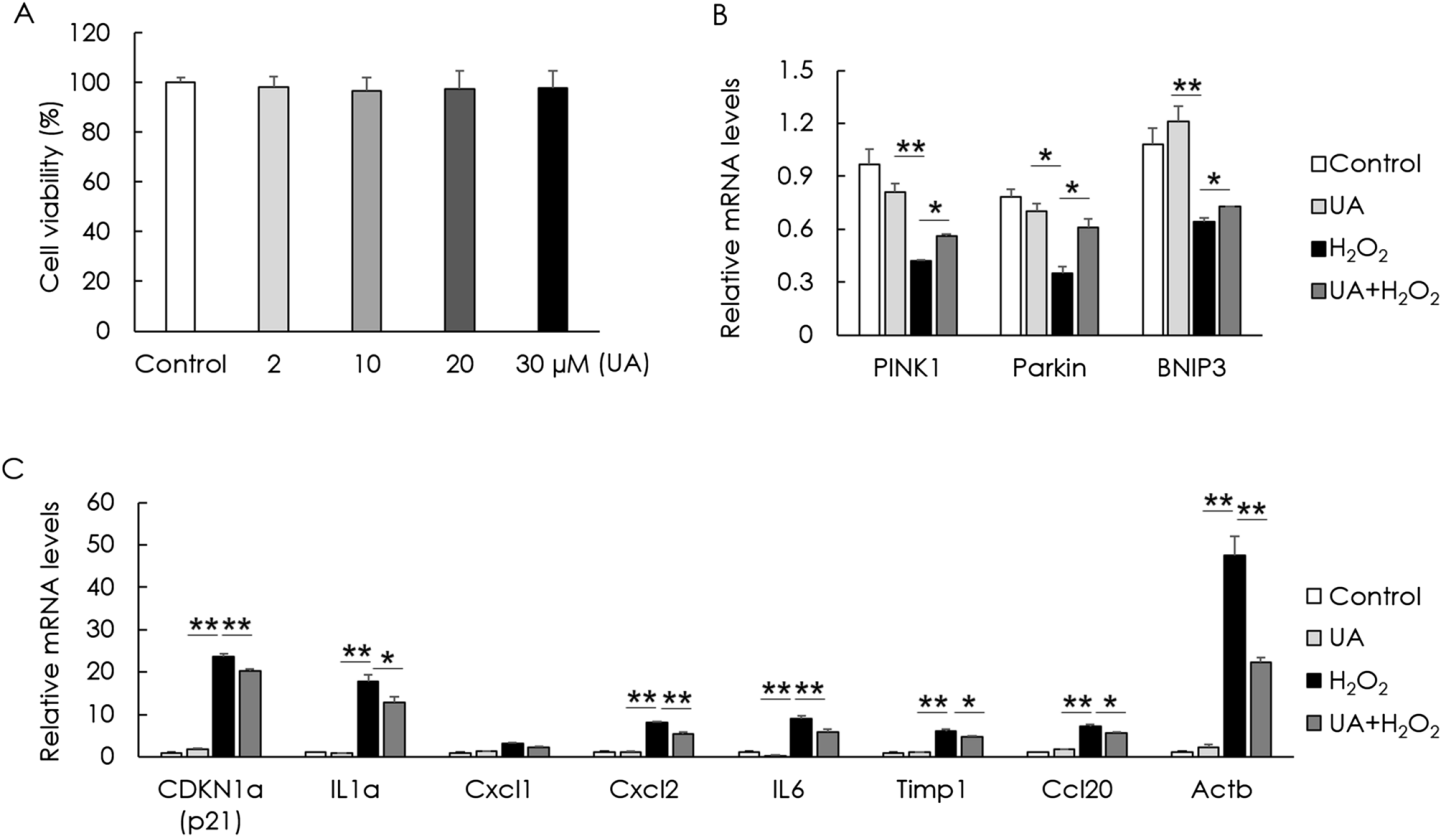
Effect of UA on gene expression levels of senescence- and mitophagy-associated molecules. HEI-OC1 cells were treated with UA at 0, 2, 10, 20, and 30 µM for 24 h. Cell viability was not affected by UA treatment (**A**). HEI-OC1 cells were pre-treated with 30 µM UA for 2 h, and then incubated with or without 2 mM H_2_O_2_ for 1 h, and incubated in culture medium for 5 days. UA pre-treatment significantly counteracted the downregulation of mitophagy-related RNAs, including those of *Pink1*, *Parkin*, and *Bnip3*, caused by H_2_O_2_ treatment of HEI-OC1 cells (**B**). UA pre-treatment significantly reduced the upregulation of senescence-associated mRNAs, including those of CDKN1a (p21), IL1a, Cxcl2, IL6, Timp1, Ccl20, and Actb, caused by H_2_O_2_ treatment of HEI-OC1 cells (**C**). Data are presented as means ± standard errors of the mean of five independent experimental results (UA, Urolithin A). *p < 0.05, **p < 0.01.

**Figure 3:**
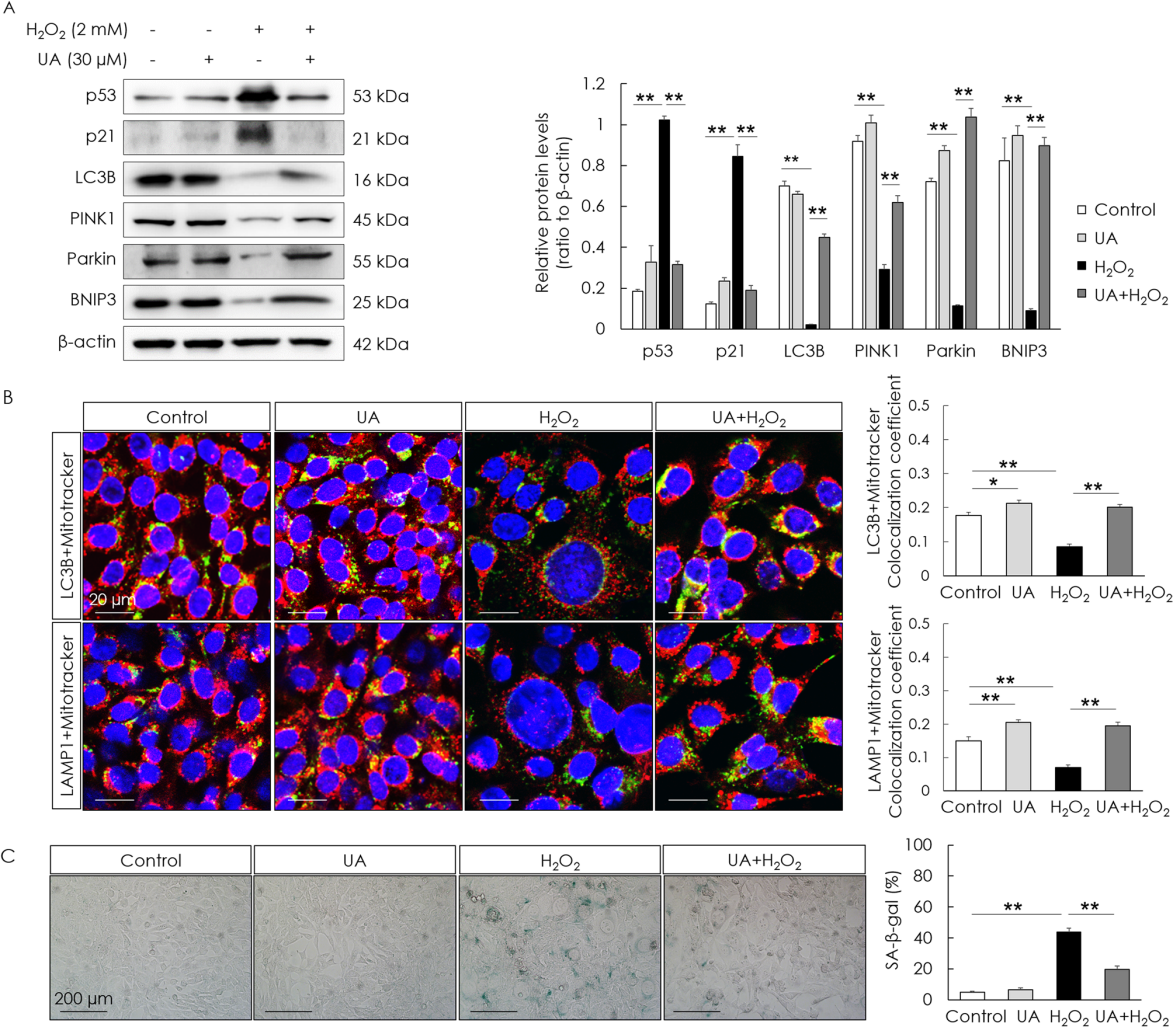
Effect of UA on levels of senescence and mitophagy in HEI-OC1 cells. HEI-OC1 cells were pre-treated with 30 µM UA for 2 h, and then incubated with or without 2 mM H_2_O_2_ for 1 h, and incubated in culture medium for 5 days. UA pre-treatment significantly decreased the levels of senescence-associated proteins (p53, p21) and significantly increased the levels of mitophagy-related proteins (PINK1, Parkin, and BNIP3) following H_2_O_2_ treatment of HEI-OC1 cells (**A**). Mitophagosomes were detected using co-localization (yellow puncta) of LC3B (green) and MitoTracker (red) (B, upper panel). Mitophagolysosomes were detected using co-localization (yellow puncta) of LAMP1 (green) and MitoTracker (red) (B, lower panel). The co-localization areas of LC3B and MitoTracker, and LAMP1 and MitoTracker were significantly reduced in H_2_O_2_-treated cells, whereas they were significantly enhanced in UA-pre-treated cells (**B**). UA pre-treatment significantly decreased the percentage of β-gal-stained cells following H_2_O_2_ treatment of HEI-OC1 cells (**C**). Data are presented as means ± standard errors of the mean of five independent experimental results (UA, Urolithin A; SA-β-gal, senescence associated-beta galactosidase). *p < 0.05, **p < 0.01. The grouping of gels/blots cropped from different parts of the same gel. Full-length blots are presented in Fig. S2.

**Figure 4:**
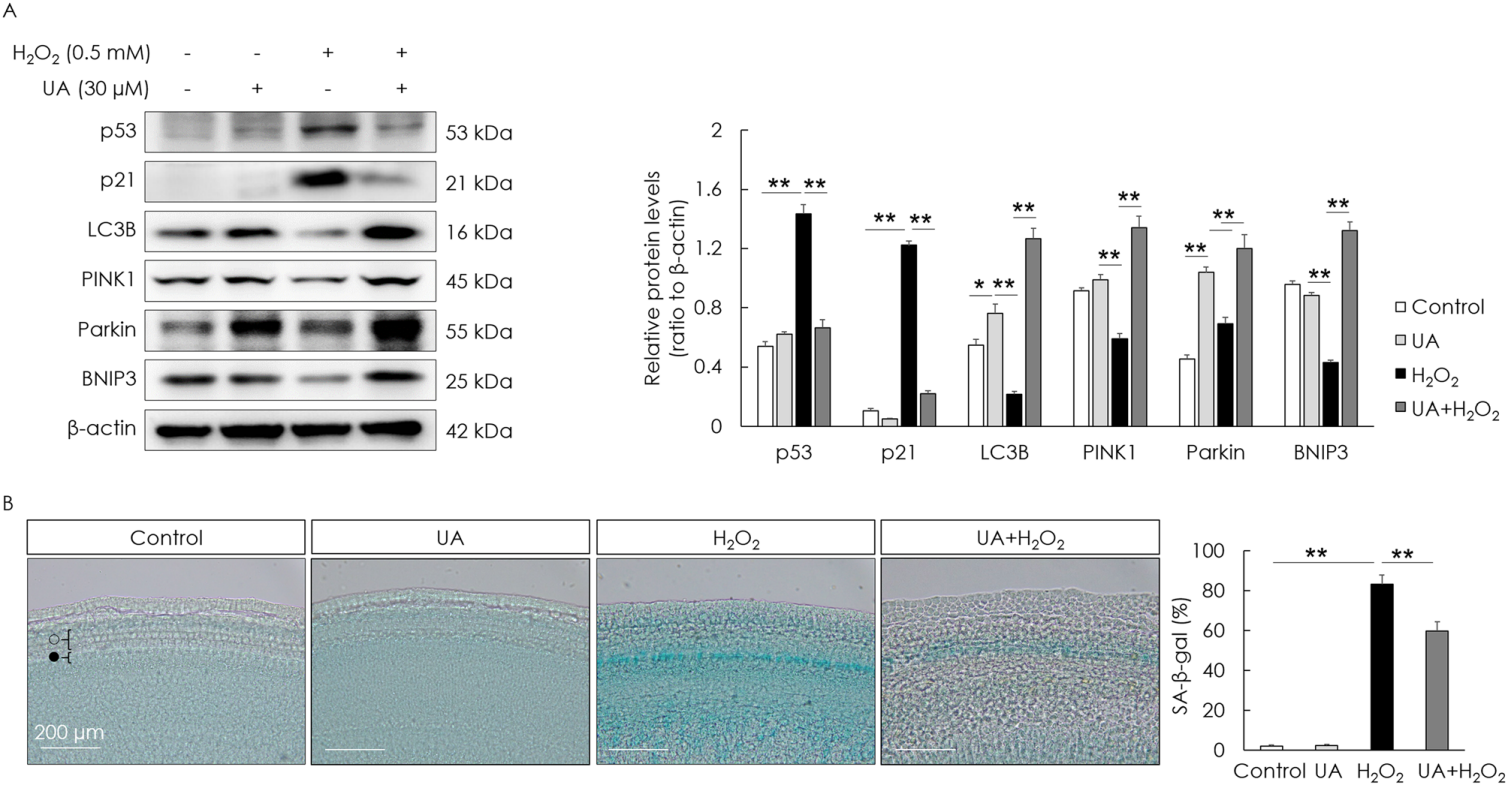
Effect of UA on levels of senescence- and mitophagy-associated molecules in cochlear explants. Cochlear explants were pre-treated with 30 µM UA for 2 h, and then incubated with 0.5 mM H_2_O_2_ for 5 h, and incubated in culture medium for 5 days. UA pre-treatment significantly decreased the levels of senescence-associated proteins (p53 and p21) and significantly increased the levels of mitophagy-related proteins (PINK1, Parkin, and BNIP3) following H_2_O_2_ treatment of cochlear explants (**A**). UA pre-treatment significantly decreased the percentage of β-gal-stained cells following H_2_O_2_ treatment of cochlear explants (**B**). Data are presented as means ± standard errors of the mean of five independent experimental results (UA, Urolithin A; ○, outer hair cells; ●, inner hair cells; SA-β-gal, senescence associated-beta galactosidase). **p < 0.01. The grouping of gels/blots cropped from different parts of the same gel. Full-length blots are presented in Fig. S3.

**Figure 5:**
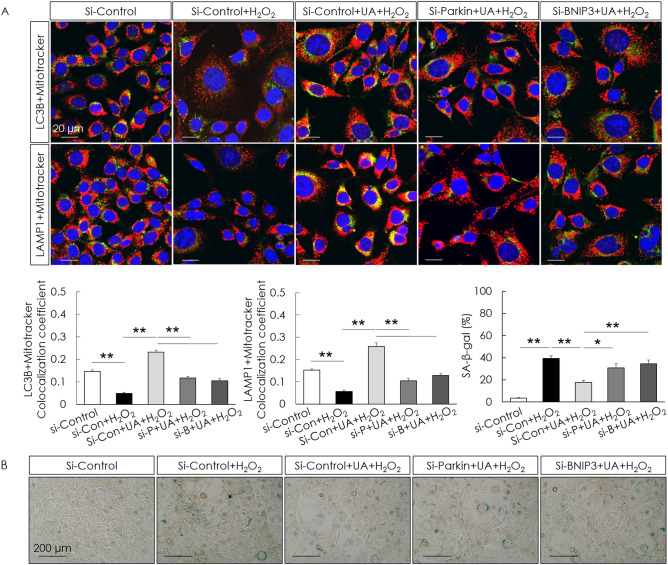
Effect of knockdown of *Parkin* and *Bnip3* on UA-induced anti-senescent activity in HEI-OC1 cells. The co-localization areas (yellow puncta) of LC3B and MitoTracker, and LAMP1 and MitoTracker were significantly increased in si-Control H_2_O_2_-treated cells upon UA pre-treatment, whereas they were significantly decreased post siRNA-mediated knockdown of *Parkin* and *Bnip3* (**A**). UA pre-treatment significantly decreased the percentage of β-gal-stained cells following H_2_O_2_ treatment of si-Control HEI-OC1 cells, whereas the percentage of β-gal-stained cells was not reduced in cells transfected with *Parkin* and *Bnip3* siRNAs (**B**). Data are presented as means ± standard errors of the mean of five independent experimental results (UA, Urolithin A; SA-β-gal, senescence associated-beta galactosidase). *p < 0.05, **p < 0.01.

To examine if UA is toxic to cells, cell viability was assessed after UA treatment. UA did not decrease the cell viability of HEI-OC1 cells (Fig. 2A). To examine the effect of UA on mitophagy and cellular senescence, senescence-associated markers and mitophagy-related genes—*Pink1*, *Parkin*, and *Bnip3*—in H_2_O_2_-induced senescent HEI-OC1 cells were examined. The gene (mRNA) expression of senescence markers was determined using the senescence-associated secretory phenotype and a classical hallmark of cellular senescence, CDKN1a (p21). Significant increases in the mRNA expression of the senescence markers, CDKN1a (p21), IL1a, Cxcl2, IL6, Timp1, Ccl20, and Actb, were detected in the H_2_O_2_-treated group. The expression of mitophagy-related genes significantly decreased in the H_2_O_2_-treated group. UA pre-treatment before H_2_O_2_ treatment significantly increased the mRNA levels of mitophagy-related genes and decreased the mRNA levels of senescence markers, compared with those in cells treated with H_2_O_2_ alone (Fig. 2B,C). UA also significantly decreased the expression of senescence-associated p53 and p21, whereas it significantly increased the expression of mitophagy-related proteins in H_2_O_2_-induced senescent HEI-OC1 cells (Fig. 3A). The formation of mitophagosomes was investigated using immunofluorescence analysis of the co-localization of LC3B and MitoTracker. This co-localization significantly decreased in H_2_O_2_-treated cells, but significantly increased upon UA pre-treatment (Fig. 3B, upper panel). The formation of mitophagolysosomes was investigated using immunofluorescence analysis of the co-localization of LAMP1, a marker of lysosomes, and MitoTracker. This co-localization significantly decreased in H_2_O_2_-treated cells, but increased upon UA pre-treatment (Fig. 3B, lower panel). The percentage of β-galactosidase-stained senescent cells reduced in H_2_O_2_-treated cells upon UA pre-treatment (Fig. 3C). UA significantly decreased the expression of senescence-associated p53 and p21, whereas it significantly increased the expression of mitophagy-related proteins, in H_2_O_2_-treated cochlear explants (Fig. 4A). The percentage of β-galactosidase-stained senescent cells reduced in H_2_O_2_-treated cochlear explants upon UA pre-treatment (Fig. 4B). To investigate whether anti-senescent activity of UA is dependent on mitophagy or by other effects, knockdown of mitophagy genes was conducted in HEI-OC1 cells. Knockdown of *Parkin* and *Bnip3* mRNAs significantly reduced the formation of mitophagosomes and mitophagolysosomes (Fig. 5A). While the percentage of β-galactosidase-stained senescent cells reduced in si-Control H_2_O_2_-treated cells upon UA pre-treatment, the percentage of β-galactosidase-stained senescent cells did not reduce post siRNA-mediated knockdown of *Parkin* and *Bnip3* (Fig. 5B). These results indicated that UA induced mitophagy and attenuated premature senescence in auditory cells.

### UA increases mitochondrial function in senescent HEI-OC1 cells

**Figure 6:**
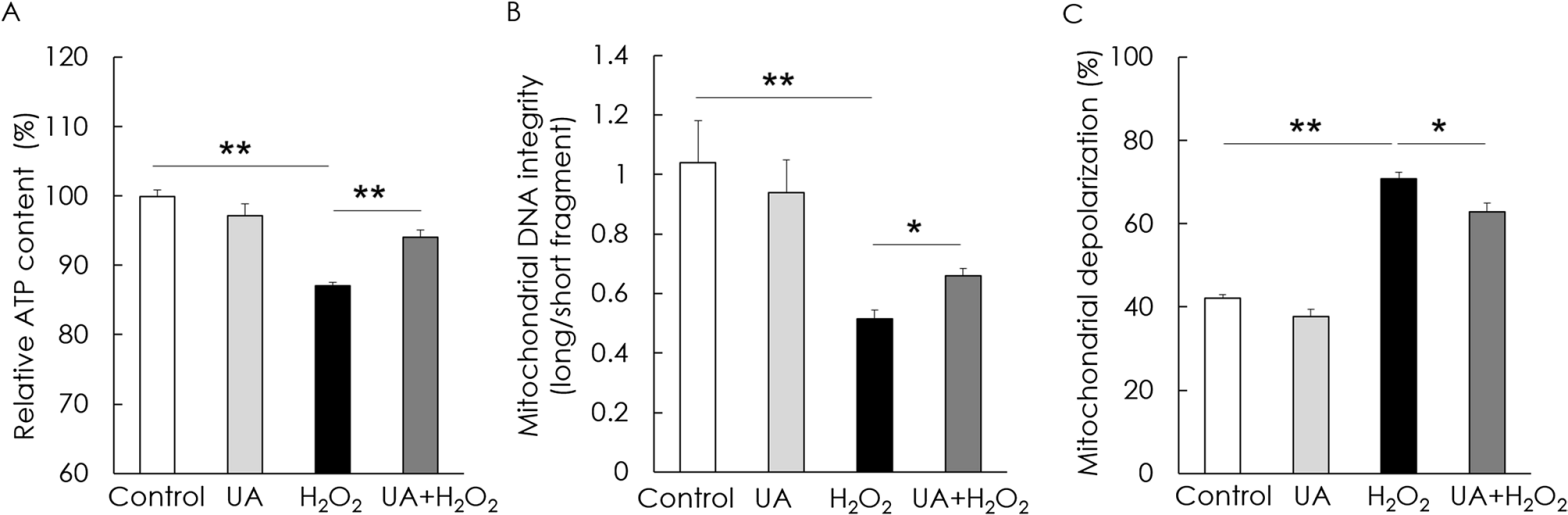
Effect of UA on ATP content, mitochondrial DNA (mtDNA) integrity, and mitochondrial depolarization in H_2_O_2_-induced senescent HEI-OC1 cells. ATP content was significantly preserved in H_2_O_2_-treated cells upon UA pre-treatment (**A**). The mtDNA integrity in the UA pre-treatment group was significantly higher than that of the H_2_O_2_ treatment group (**B**). Mitochondrial depolarization significantly decreased in UA-pre-treated cells as compared to that in H_2_O_2_-treated cells (**C**). Data are presented as means ± standard errors of the mean of five independent experimental results (UA, Urolithin A). *p < 0.05, **p < 0.01.

ATP content, mtDNA integrity, and mitochondrial depolarization were examined in H_2_O_2_-induced senescent HEI-OC1 cells to investigate the beneficial effect of UA on mitochondrial function. The ATP content in the H_2_O_2_-treated group was significantly lower than that in the control group; however, pre-treatment with UA significantly increased the ATP content following H_2_O_2_ treatment (Fig. 6A). mtDNA integrity was assessed as the ratio of long mtDNA to short mtDNA. The mtDNA integrity of the H_2_O_2_ treatment group was significantly lower than that of the control group; however, the mtDNA integrity of the UA pre-treatment before H_2_O_2_ treatment group was significantly higher than that of the H_2_O_2_ treatment group (Fig. 6B). Mitochondrial membrane potential is a parameter necessary for functional mitochondria. The mitochondrial depolarization values of the H_2_O_2_ treatment group were significantly higher than those of the control group. However, UA pre-treatment significantly decreased the mitochondrial depolarization values following H_2_O_2_ treatment (Fig. 6C). These results indicated that UA increased the mitochondrial integrity and function in senescent auditory cells.

## Discussion

Mitochondria generate ATP in cells and are important for energy supply. They are also critical for neurotransmission in the auditory system^15^. The numbers of damaged and dysfunctional mitochondria increase with age, triggering the production of reactive oxygen species and the consequent age-dependent decline in organ function^16,17^. Mitophagy maintains a healthy mitochondrial population through the efficient removal of damaged mitochondria, and contributes to mitochondrial quality control^18^.

In this study, oxidative stress-induced premature senescence was associated with a progressive decrease in the expression of mitophagy-related genes and proteins. The PINK1/Parkin pathway is a major pathway of mitophagy for mitochondrial quality control in mammalian cells. PINK1 recruits Parkin upon the loss of mitochondrial membrane potential. This subsequently activates the ubiquitin–proteasome system and promotes the engulfment of damaged mitochondria through the formation of mitophagosomes^19^. BNIP3 is a key factor in Parkin-independent regulation of mitophagy^20^. It binds to LC3 on autophagosomes to promote the autophagic engulfment of mitochondria^4^. The decrease in the expression of mitophagy-related genes and proteins (PINK1, Parkin, and BNIP3) in premature senescent cells indicated a decline in mitophagy, and the accumulation of damaged mitochondria.

Competent mitochondrial activity is highly associated with mtDNA integrity and ATP production^21^. Through the present study, we have provided evidence that UA restores mitochondrial integrity and function, including ATP production and mitochondrial membrane potential, in senescent auditory cells. Damaged mitochondria are engulfed by autophagosomes, which subsequently fuse with lysosomes and form mitophagolysosomes during mitophagy^22^. Our results show that UA restored the levels of mitophagosomes and mitophagolysosomes in senescent auditory cells.

Mitochondria-targeted therapeutic strategies for pathologies associated with aging have received attention due to the effect of enhancing mitochondrial function^12^. Mitophagy maintains neuronal function through maintaining a healthy mitochondrial population and efficient energy supply in the nervous system^23^. Mitophagy inducers such as Kaempferol and Rhapontigenin inhibited memory loss in nematode and rodent models of Alzheimer’s disease through the elimination of dysfunctional mitochondria^24^. Induction of mitophagy through NAD^+^ augmentation forestalled Alzheimer’s disease, Parkinson’s disease, and Huntington disease in animal and cell culture models^25^. In contrast, inactivation of PINK1 resulted in neuronal degeneration and Huntington disease^26^.

UA has previously been reported to exert anti-aging effects in several cells^27^. UA improves mitochondrial respiratory capacity and induces the expression of mitochondrial oxidative phosphorylation proteins^11^. It also exhibits multiple biological activities, including antioxidant, anti-inflammatory, anticancer, and antimicrobial properties^28,29^. The biological effects of UA in neurodegeneration and diseases of the central nervous system have been reported^30^. UA improved associative memory and neuronal survival through the induction of PINK1 expression in neurons of an Alzheimer’s disease mouse model^31^. UA also protected against ischemic stroke induced by occlusion of the cerebral artery^32^. In addition, UA exhibited neuroprotective effects in a mouse model of multiple sclerosis by reducing white matter demyelination^33^.

It has been reported that UA supplementation in humans is suitable, in terms of safety and bioavailability, to improve mitochondrial and cellular health. Nutritional intervention with UA can be applied to humans for promoting healthy mitochondrial function throughout their lifetimes^12^.

Our results demonstrated that UA counteracted mitophagy decline and maintained mitochondrial function in premature senescent auditory cells. UA administration might be a promising strategy for preventing a decline in mitochondrial function in auditory cells, and age-related hearing loss.

## Conclusion

This study proved that UA induces mitophagy and prevents premature senescence in auditory cells. The activation of mitophagy using UA can be a potential preventive strategy for patients with age-related hearing loss.

## Materials and methods

### Cell culture and treatment

Low-dose H_2_O_2_ has been used to induce premature senescence in auditory cells^34,35^. HEI‐OC1 cells were cultured in high-glucose Dulbecco’s modified Eagle’s medium (DMEM; Welgene, Daegu, Korea) supplemented with 10% fetal bovine serum (FBS; Lonza Walkersville, MD, USA) at 33 °C under a humidified atmosphere with 5% CO_2_. The cells were treated with 2 mM H_2_O_2_ (Daejung Chemicals, Siheung, Korea) for 1 h to induce a senescent phenotype^30^. Cell viability was detected using the EZ-Cytox cell viability assay kit (Daeil Lab Service, Seoul, Korea), according to the manufacturer’s protocol. The cells were pre-treated with 30 µM UA (Sigma, MO, USA) for 2 h, and then incubated with 2 mM H_2_O_2_ for 1 h. After washing with PBS, the cells were incubated in culture medium for 5 days.

### Mice and animal care

C57BL/6 J mice were purchased from the Animal Facility of Aging Science, KBSI Gwangju Center (Gwangju, Korea). The animal experiments were approved by the Chosun University Institutional Animal Care and Use Committee (approval no. CIACUC2020-A0002). All experiments were performed in accordance with the ARRIVE guidelines and strict regulations approved by the committee. The mice were euthanized by cervical dislocation under 5% isoflurane anesthesia. Cochleae and organ of Corti explants were isolated from postnatal day 4 mice for further studies. Each organ of Corti explant was pre-treated with 30 µM UA for 2 h, exposed to 0.5 mM H_2_O_2_ for 5 h, replaced in fresh culture medium, and incubated for 1, 3, and 5 days.

### Senescence-associated $\upbeta$-galactosidase activity assay

Senescence-associated β-galactosidase activity was measured using the Senescence β-Galactosidase Staining Kit (Cell Signaling Technology, MA, USA), according to the manufacturer’s protocol. Briefly, cultured and treated cells in a 6-well plate were washed twice with PBS and fixed in 4% paraformaldehyde in PBS for 15 min. After washing twice with PBS, 1 mL staining solution was added to each well, and the plate was sealed with parafilm, incubated at 37 °C overnight (no CO_2_), and imaged using an ECLIPSE Ti2-E microscope (Nikon, Tokyo, Japan). Cultured and treated cochlear explants (the whole basilar membrane containing the organ of Corti) were washed twice with PBS and fixed overnight in 4% paraformaldehyde in PBS. After washing twice with PBS, the samples were incubated for 2 h at 37 °C (no CO_2_) with 200 µL staining solution, washed with PBS, and mounted on microscope slides with mounting medium (Biomeda Corp., CA, USA). The specimens were analyzed and scanned with a LEICA ICC50 E microscope (Leica Microsystems, Heerbrugg, Switzerland).

### Cell population doubling rate

To confirm the population doubling rate of HEI-OC1 cells, cell numbers were counted using a hemocytometer; 1 × 10^5^ cells were plated in a 6-well plate, pre-treated (control and 2 mM H_2_O_2_), replaced in normal culture medium, and then incubated for an interval (3 days). The cells were then harvested, counted, and replated. To evaluate the population doubling rate, the numbers were converted according to the following formula: population doubling level (PDL) = log (*N*_*f*_* / N*_*o*_) / log2, where *N*_*f*_ is the final cell number and *N*_*o*_ is the initial number of seeded cells.

### Measurement of ATP contents

The ATP levels were measured using an ATP Assay Kit (BioVision, CA, USA), following manufacturer’s protocol. The absorbance of samples and standards was measured with a BioTek ELz800 microplate reader (BioTek, VT, USA). Intracellular ATP levels were determined based on a standard curve and calculated as nmol/µL (per 10^5^ cells) and expressed as a relative percentage of that of the control.

### Mitochondrial DNA damage

HEI-OC1 cells were seeded in a 6-well plate. DNA was isolated using an AccuPrep Genomic DNA Extraction Kit (Bioneer, Daejeon, Korea). The total DNA (20 ng) was assessed using two set pairs of primers with a 10.1 kb and 117 bp amplicon (a short amplicon was used as a normalization factor of mtDNA copies).

### Measurement of mitochondrial membrane potential

HEI-OC1 cells were washed once with PBS and stained with 200 nM MitoTracker Red CMXRos (Thermo Fisher Scientific, MA, USA) for 20 min in Opti-MEM Reduced Serum Medium (Thermo Fisher Scientific). The cells were then washed twice with PBS, harvested, used for flow cytometry (BD Biosciences, CA, USA), and analyzed using Cell Quest software (BD Biosciences).

### Quantitative real-time PCR

Total cellular RNA was isolated using Hybrid-RTM (GeneAll Biotechnology, Seoul, Korea), following the manufacturer’s instructions. The extracted total RNA (5 μg) was reverse-transcribed into cDNA using the M-MLV cDNA Synthesis Kit (Enzynomics, Daejeon, Korea). qRT-PCR analysis was performed using the SYBR Premix Ex Taq kit (Takara Bio, Shiga, Japan). The cDNA was amplified with the following primers: mouse *Pink1*, 5ʹ-GCTTGCCAATCCCTTCTATG-3ʹ and 5ʹ-CTCTCGCTGGAGCAGTGAC-3ʹ^36^; mouse *Parkin*, 5ʹ-AAACCGGATGAGTGGTGAGT-3ʹ and 5ʹ-AGCTACCGACGTGTCCTTGT-3ʹ^37^; mouse *Bnip3*, 5ʹ-GCTCCCAGACACCACAAGAT-3ʹ and 5ʹ-TGAGAGTAGCTGTGCGCTTC-3ʹ^38^; mouse *Cdkn1a* (p21), 5ʹ-GCA GAT CCA CAG CGA TAT CCA-3ʹ and 5ʹ-AAC AGG TCG GAC ATC ACC AG-3ʹ; mouse *Il1a*, 5ʹ-AGG GAG TCA ACT CAT TGG CG-3ʹ and 5ʹ-TGG CAG AAC TGT AGT CTT CGT-3ʹ; mouse *Cxcl1*, 5ʹ-ACC GAA GTC ATA GCC ACA CTC-3ʹ and 5ʹ-CTC CGT TAC TTG GGG ACA CC-3ʹ; mouse *Cxcl2*, 5ʹ-CCC AGA CAG AAG TCA TAG CCA C-3ʹ and 5ʹ-TGG TTC TTC CGT TGA GGG AC-3ʹ; mouse *Il6*, 5ʹ-TGA GAA AAG AGT TGT GCA ATG G-3ʹ and 5ʹ-GGT ACT CCA GAA GAC CAG AGG-3ʹ; mouse *Timp1*, 5ʹ-CAC ACC AGA GCA GAT AC CATG A-3ʹ and 5ʹ-GGG GAA CCC ATG AAT TTA GCC-3ʹ; mouse *Actb*, 5ʹ-GTC CAC ACC CGC CAC C-3ʹ and 5ʹ-ACC CAT TCC CAC CAT CAC AC-3ʹ^39^; mouse *Ccl20*, 5ʹ-AAC TGG GTG AAA AGG GCT GT-3ʹ and 5ʹ-GTC CAA TTC CAT CCC AAA AA-3ʹ^40^; and mouse *Gapdh*, 5ʹ-GTATTGGGCGCCTGGTCACC-3ʹ and 5ʹ-CGCTCCTGGAAGATGGTGATGG-3ʹ^41^. The expression of the target gene was normalized to that of *Gapdh*. The expression of the target gene was calculated using the 2^−ΔΔCt^ method and expressed as fold change over that of the control.

### Western blot analysis

Cells were lysed in lysis buffer containing 20 mM HEPES (pH 7.4), 2 mM EGTA, 50 mM glycerol phosphate, 1% Triton X-100, 10% glycerol, 1 mM dithiothreitol, 1 mM phenylmethylsulfonylfluoride, 10 μg/mL leupeptin, 10 μg/mL aprotinin, 1 mM Na_3_VO_4_, and 5 mM NaF. Proteins were extracted by centrifugation, and equal amounts of proteins were loaded onto a gel, resolved by SDS-PAGE, and transferred onto a PVDF membrane (Millipore, MA, USA). The membranes were blocked for 1 h with 5% skim milk in 1 × TBS-T (20 mM Tris–HCl, 137 mM NaCl, pH 7.5, and 0.1% Tween-20). The membranes were probed overnight with a primary antibody at 4 °C. The following primary antibodies were used: p53, p21 (ABclonal Technology, MA, USA), LC3B (Cell Signaling Technology), PINK1, Parkin, BNIP3 (Thermo Fisher Scientific), β-actin (Santa Cruz, CA, USA). The primary antibody was detected for 2 h with anti-mouse in sheep and anti-rabbit in donkey (Jackson ImmunoResearch, PA, USA). Protein bands were visualized using a western blot detection system (Millipore) and developed using an image analyzer (Vilber, Collégien, France). Protein levels were quantitated with Image J (NIH, MD, USA).

### Immunofluorescence analysis

For mitochondrial staining, cells were stained with the mitochondrial probe, MitoTracker Red CMXRos (Thermo Fisher Scientific), according to the manufacturer’s protocol. The stained cells were washed twice with PBS and fixed in 4% paraformaldehyde for 15 min. The fixed cells were washed twice with PBS, blocked in 1% BSA solution, and incubated overnight with LC3B (Cell signaling Technology) and LAMP1 (Santa Cruz Biotechnology, TX, USA) at 4 °C. The cells were then washed twice with PBS, incubated with FITC-conjugated secondary antibodies (chicken anti-mouse and goat Alexa Fluor 488; Invitrogen, MA, USA) for 2 h, and again washed twice with PBS. Thereafter, the cells were washed with PBS and Fluorescent Mounting Medium with DAPI (GBI Labs, WA, USA), and then imaged by confocal microscopy (Carl Zeiss, Oberkochen, Germany) with Zeiss microscope image software ZEN (Carl Zeiss). The co-localization coefficient levels were quantified with Image J (NIH).

### Transfection of siRNAs

HEI-OC1 cells (1 × 10^5^ per well) were cultured in 6-well plates for 24 h, and then transfected with si-Control, si-Parkin, and si-BNIP3 (Santa Cruz Biotechnology, TX, USA) for 24 h using Lipofectamine RNAiMAX reagent (Invitrogen, CA, USA) according to the manufacturer’s protocol. The transfected cells were pre-treated with 30 µM UA (Sigma, MO, USA) for 2 h, and then incubated with 2 mM H_2_O_2_ for 1 h. After washing with PBS, the cells were incubated in culture medium for 5 days.

### Statistical Analyses

All statistical analyses were performed with SPSS 25.0 software (SPSS Inc., IL, USA). Data were analyzed by one-way analysis of variance, followed by Tukey’s honestly significant difference post-hoc test. Senescent cell and population doubling levels were compared between groups using Student’s *t*-test. The differences were considered statistically significant when the *p*-value was less than 0.05.

## Supplementary Information

Supplementary Information.

## Data Availability

The datasets generated and analysed during the current study are available in the Mendeley Data repository, https://data.mendeley.com/datasets/ft3dsdh59x/1.
