# Supplementary material for: Urolithin A attenuates auditory cell senescence by activating mitophagy
Source: Sci Rep. 2022 May 11;12:7704. doi: 10.1038/s41598-022-11894-2 (PMC9095590; doi:10.1038/s41598-022-11894-2)
Supplement: Supplementary file 1 — Supplementary Information. [file 41598_2022_11894_MOESM1_ESM.pdf]

Urolithin A attenuates auditory cell senescence by activating mitophagy

Sung Il Cho[1], Eu-Ri Jo[1], Hansoo Song[2]

Department of Otolaryngology-Head and Neck Surgery[1], Department of Occupational & Environmental Medicine[2], Chosun University College of Medicine, Gwangju, South Korea

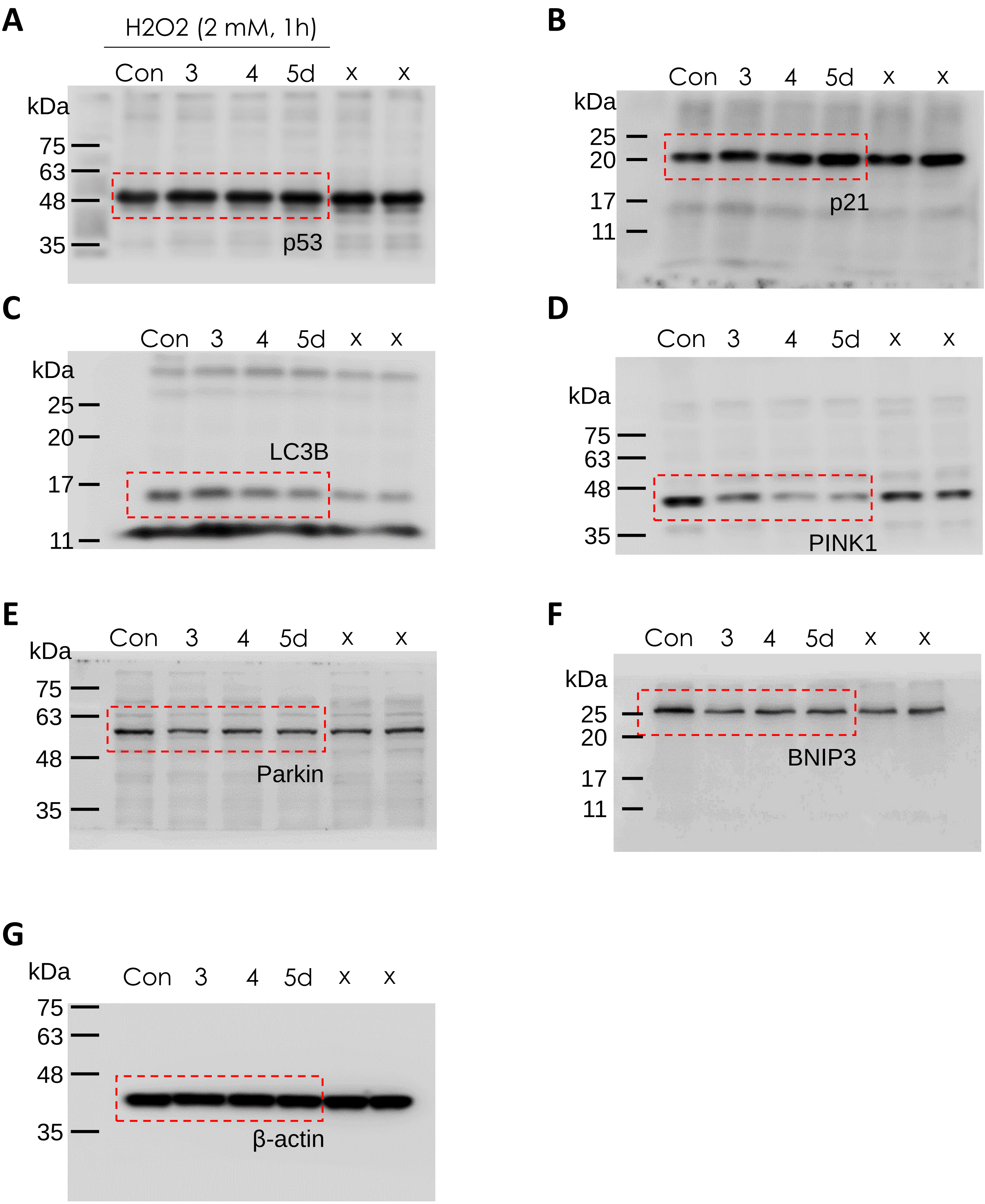

Supplementary Figure S1A. Full-length blots for Figure 1A. Red boxes surround the cropped portions of each blot displayed in the Figure 1A. (A) Antibody: p53, SDS-PAGE 12% gel. (B) Antibody: p21, SDS-PAGE 12% gel. (C) Antibody: LC3B, SDS-PAGE 12% gel. (D) Antibody: PINK1, SDS-PAGE 12% gel. (E) Antibody: Parkin, SDS-PAGE 12% gel. (F) Antibody: BNIP3, SDS-PAGE 12% gel. (G) Antibody: β-actin, SDS-PAGE 12% gel.

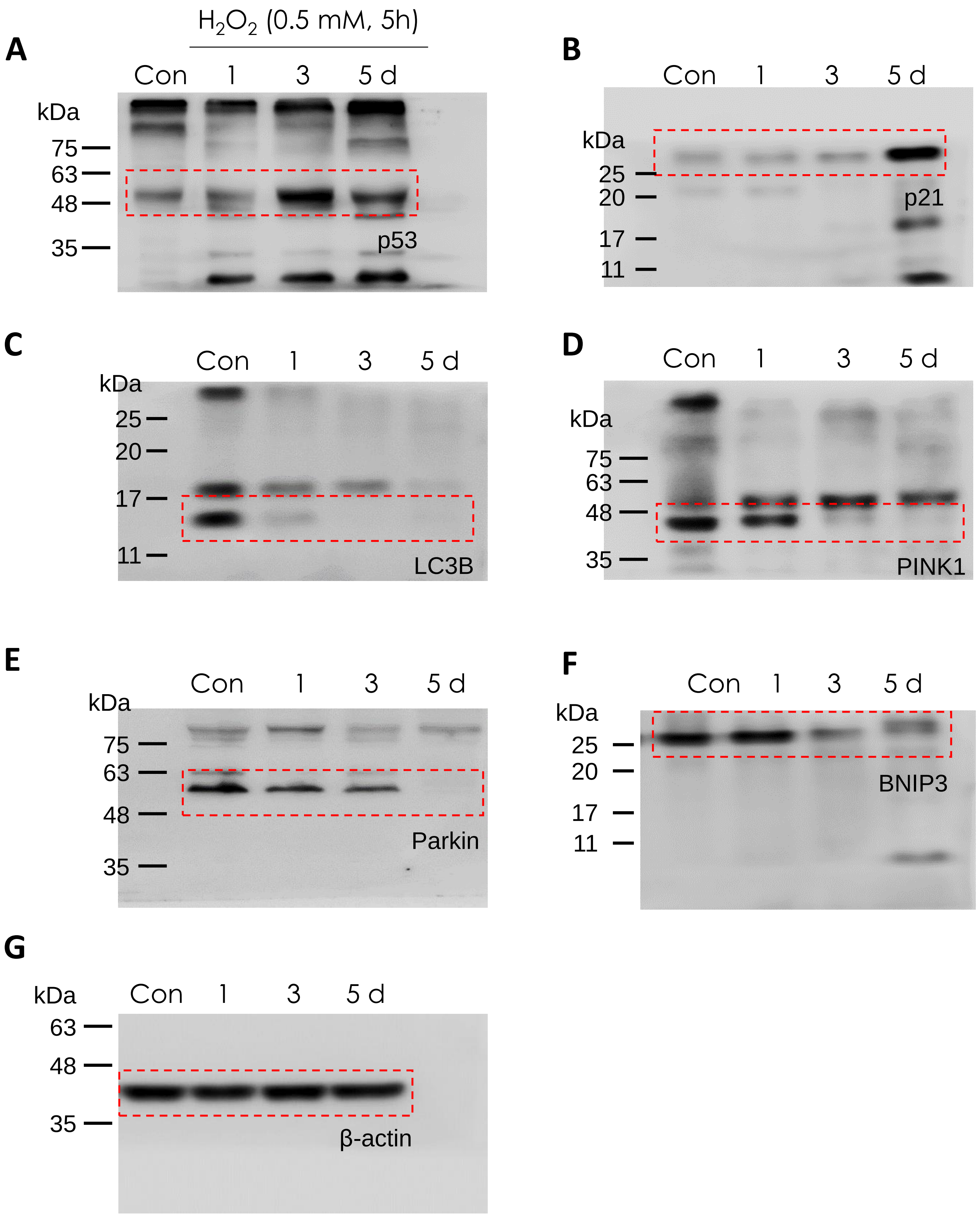

Supplementary Figure S1B. Full-length blots for Figure 1B. Red boxes surround the cropped portions of each blot displayed in the Figure 1B. (A) Antibody: p53, SDS-PAGE 12% gel. (B) Antibody: p21, SDS-PAGE 12% gel. (C) Antibody: LC3B, SDS-PAGE 12% gel. (D) Antibody: PINK1, SDS-PAGE 12% gel. (E) Antibody: Parkin, SDS-PAGE 12% gel. (F) Antibody: BNIP3, SDS-PAGE 12% gel. (G) Antibody: β-actin, SDS-PAGE 12% gel.

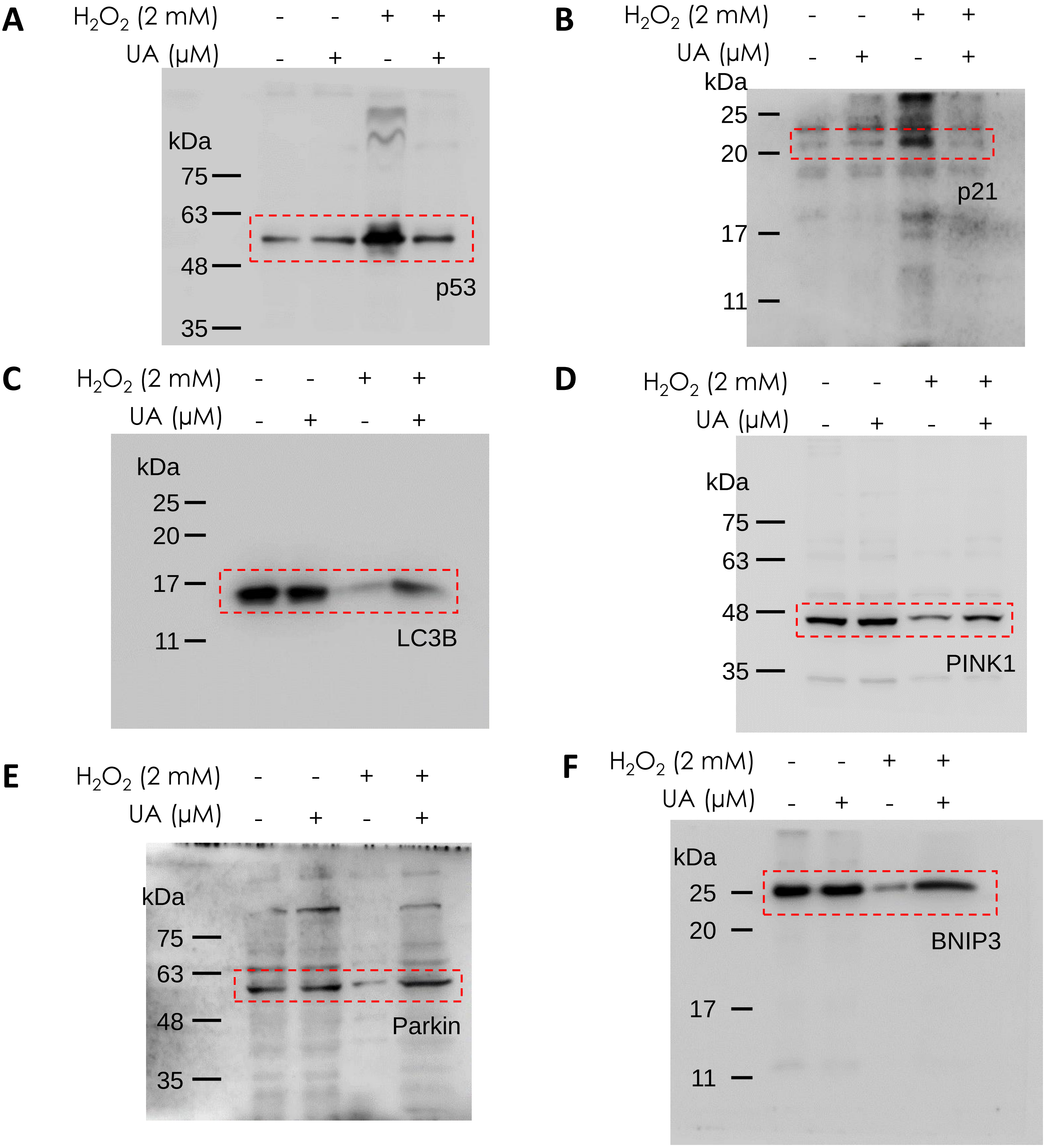

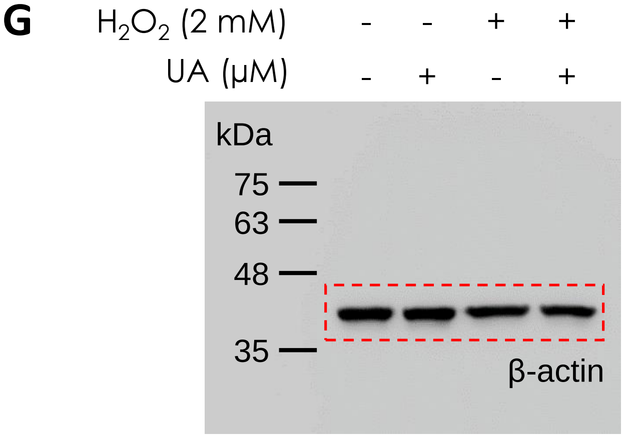

Supplementary Figure S2. Full-length blots for Figure 3A. Red boxes surround the cropped portions of each blot displayed in the Figure 3A. (A) Antibody: p53, SDS-PAGE 12% gel. (B) Antibody: p21, SDS-PAGE 12% gel. (C) Antibody: LC3B, SDS-PAGE 12% gel. (D) Antibody: PINK1, SDS-PAGE 12% gel. (E) Antibody: Parkin, SDS-PAGE 12% gel. (F) Antibody: BNIP3, SDS-PAGE 12% gel. (G) Antibody: β-actin, SDS-PAGE 12% gel.

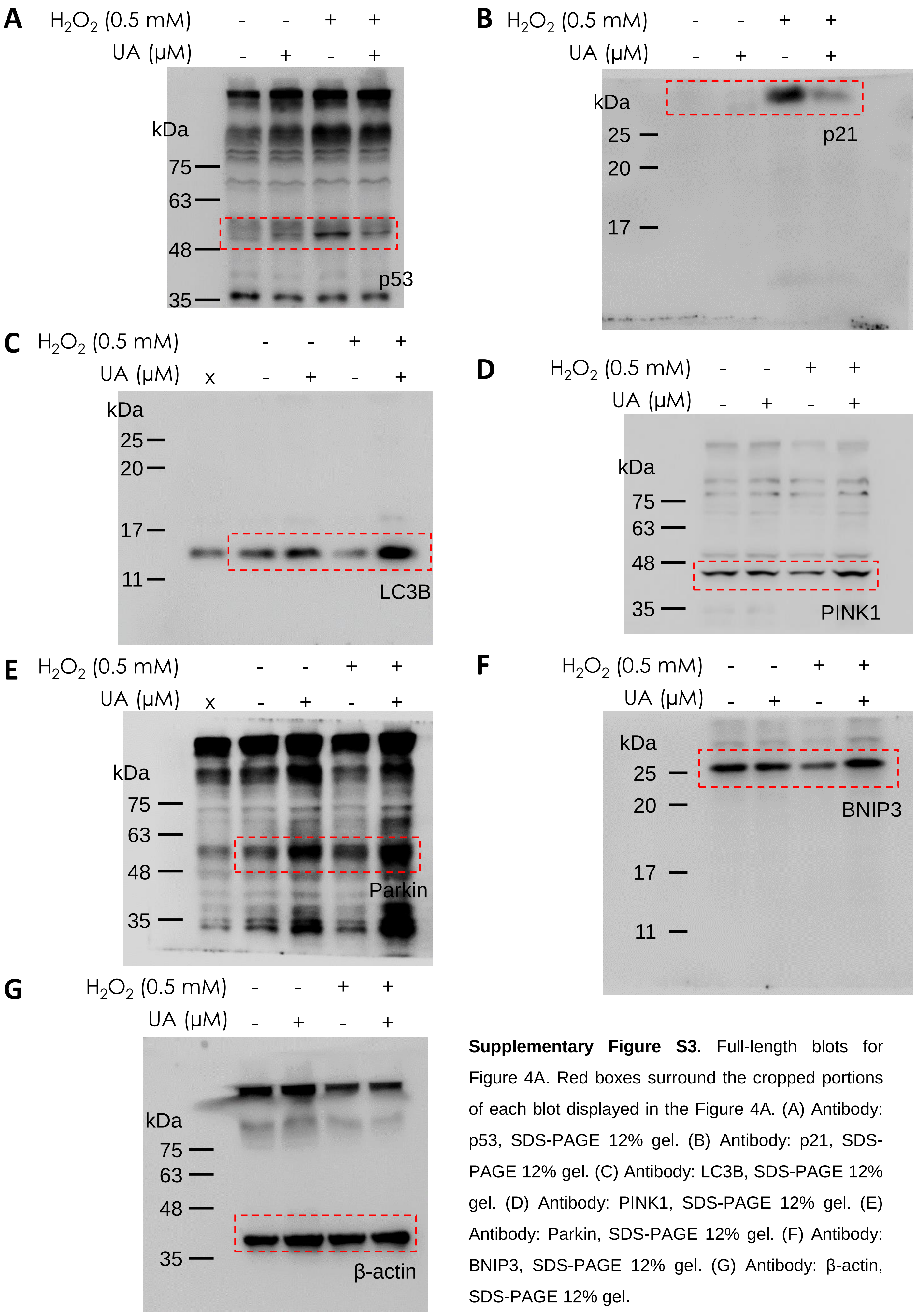

Supplementary Figure S3. Full-length blots for Figure 4A. Red boxes surround the cropped portions of each blot displayed in the Figure 4A. (A) Antibody: p53, SDS-PAGE 12% gel. (B) Antibody: p21, SDS-PAGE 12% gel. (C) Antibody: LC3B, SDS-PAGE 12% gel. (D) Antibody: PINK1, SDS-PAGE 12% gel. (E) Antibody: Parkin, SDS-PAGE 12% gel. (F) Antibody: BNIP3, SDS-PAGE 12% gel. (G) Antibody: β-actin, SDS-PAGE 12% gel.
